# Management of colorectal anastomotic stricture with multidiameter balloon dilation: long-term results

**DOI:** 10.1007/s10151-020-02318-2

**Published:** 2020-08-05

**Authors:** R.-H. Chan, S.-C. Lin, P.-C. Chen, W.-T. Lin, C.-H. Wu, J.-C. Lee, B.-W. Lin

**Affiliations:** Division of Colorectal Surgery, Department of Surgery, National Cheng Kung University Hospital, College of Medicine, National Cheng Kung University, 138 Sheng-Li Road, Tainan, 704 Taiwan

**Keywords:** Colorectal anastomotic stricture, Multidiameter balloon dilation, Anastomosis, Surgical, Dilatation, Endoscopy

## Abstract

**Background:**

Postoperative colorectal anastomotic strictures are quite common. As such, many techniques have been available to address such a problem, one of which is endoscopic dilation. The aim of the present study was to evaluate the long-term outcomes following endoscopic dilation using a multidiameter balloon.

**Methods:**

A retrospective study was conducted on patients with postoperative anastomotic stenosis treated with endoscopic dilation using a multidiameter balloon at our institution, in January 2005–December 2019 were retrospectively reviewed, excluding those with tumor recurrence. Perioperative factors, complications, and recurrence rates were analyzed.

**Results:**

There were 40 patients, (22 males and 18 females, mean age 64.6 ± 10.7 years, range 33–84 years). The median follow-up period was 56 months (interquartile range 22.5–99 months). Only 1 complication occurred, micro-perforation due to guided wire injury, which was managed conservatively. Five (12.5%) patients developed restenosis and underwent repeat balloon dilation. None of the five recurrences required more aggressive management, such as redo anastomosis.

**Conclusions:**

Endoscopic multidiameter balloon dilation is a safe and effective method for treating benign colorectal anastomotic strictures.

## Introduction

The occurrence of benign anastomotic strictures after colorectal surgery is not uncommon [1] with studies showing an incidence rate of 3–30% [2, 3]. Possible causes for such a condition include several factors, such as radiation, anastomotic ischemic changes, anastomotic leakage, and anastomotic technique [4–6]. A stricture is defined as the narrowing of the intestine or obstruction in the flow of its contents that result in clinical symptoms of complete or partial bowel obstruction [7]. An endoscopic description may be used to characterize strictures according to the inability to pass a 12-mm diameter sigmoidoscope through the narrowing lumen [2]. Most strictures involve the middle and lower rectum and may spontaneously improve without treatment [8]. However, persist strictures would require intervention, such as endoscope- or fluoroscope-guided dilation, stenting, redo anastomosis, transanal stricturoplasty, electrocautery resection, or incision with or without dilation [9]. Among the aforementioned methods, endoscopic balloon dilation has been proven safe and effective for the treatment of benign strictures of colorectal anastomosis [9, 10]. A recent systematic review estimated immediate success rates of approximately 86, with 58% of the patients achieving long-term clinical efficacy [11]. The present study therefore investigated the short-term complications and long-term outcomes of multidiameter balloon dilation for benign colorectal anastomotic strictures.

## Materials and methods

Medical records were reviewed to identify patients at our institution diagnosed with an anastomotic stricture in January 2005–December 2019. This study was approved by the Institutional Review Board, National Cheng Kung University Hospital with IRB No. A-ER-109-085.

The diagnosis was based on colonoscopy findings revealing a less than 12-mm diameter lumen and inability of the scope to pass. All patients underwent abdominal computed tomography scan to exclude other causes of lumen stricture. Patients with tumor recurrence and extra-luminal compression were excluded. Those who had strictures located within 8 cm above the anal verge (AAV) had finger dilation followed by endoscopic balloon dilation if finger dilation failed. Patients who had a diverting ostomy had colonoscopy before reversal surgery. Anastomotic strictures were treated first, once identified, after which ostomy reversal surgery was performed.

**Fig. 1:**
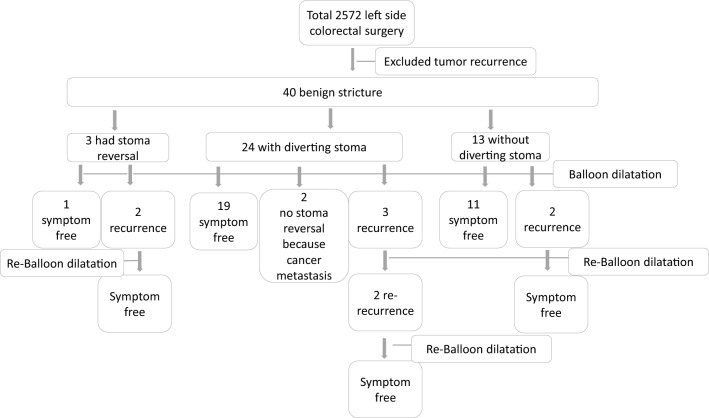
STROBE flowchart. Left side colorectal surgery included left hemicolectomy, anterior resection, low anterior resection, total mesorectal excision, and inter-sphincter resection

The medical records of the patients included in the study whom were retrospectively reviewed for sex, age, etiology of previous surgeries, types of colorectal surgery, surgical anastomosis technique, regional radiotherapy, and postoperative complications. A STROBE flowchart is provided (Fig. 1) short-term outcomes included perioperative data related to the balloon dilation procedure, including duration, number of sessions, and complications, while long-term outcomes included data collected during the follow-up period and data regarding recurrence.

Fisher's exact test was used to determine variables associated with stricture recurrence. Recurrence was defined as symptom recurrence or follow-up colonoscopy findings again revealing the inability of the scope to pass through anastomotic site.

A CRE™ wire-guided multidiameter balloon dilator (Microvasive, Boston Scientific Corp., Natick, MA, USA) was used for all procedures. A successful procedure was defined as the achievement of an anastomotic lumen wide enough to allow passage of a standard 13-mm diameter colonoscope after dilation.

### Technique for balloon dilation

**Fig. 2:**
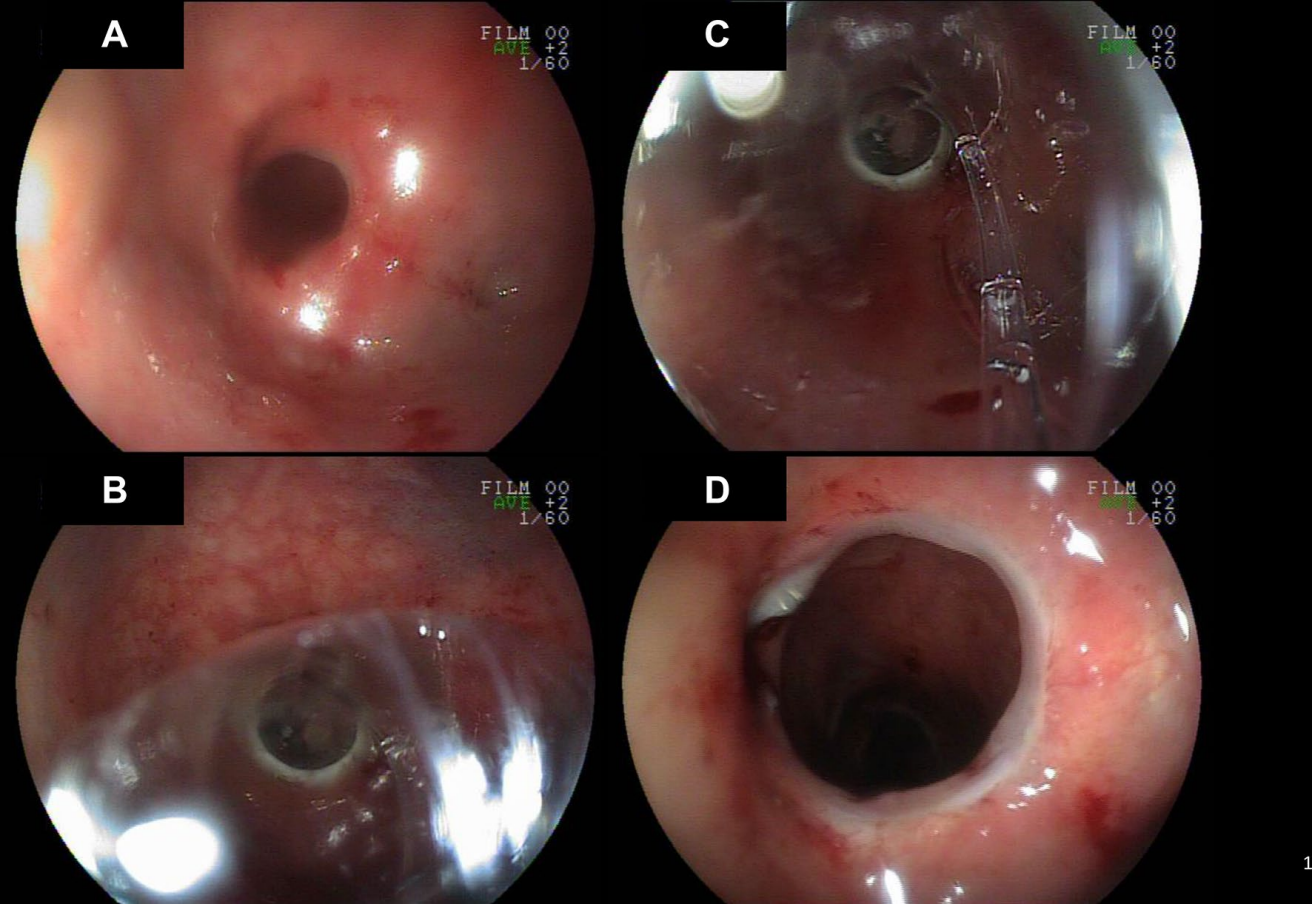
Balloon dilation. **a** The stricture site was identified through colonoscopy. **b** The CRE™ wire-guided balloon dilator was inserted through the working channel of the colonoscope and passed the stricture site. **c** The balloon was filled with distilled water to maintain the outer diameter. **d** After dilation, the stricture widened to allow scope passage

All procedures were performed under sedation. All patients had routine bowel preparation prior to colonoscope-guided balloon dilation. Patients were positioned in the left lateral decubitus position or lithotomy position as preferred by the physician. The stricture site was identified using a colonoscope. The tip of the a CRE™ (controlled radial expansion) multidiameter wire-guided balloon dilator, which had a balloon length of 5.5 cm, was inserted through the working channel of the colonoscope and passed 2–3 cm beyond the stricture site. The balloon was filled with distilled water or air to maintain an outer diameter of 12 mm and was kept in the same position for 5 min using the standard inflation pressure (3 atm) suggested by the manufacturer. Thereafter, the balloon was deflated and withdrawn below the stricture site. The catheter was reapplied at the same site with the balloon being re-inflated to keep the outer diameter at 13.5 mm (at 4.5 atm) for another 5 min. The same procedure was repeated with the balloon outer diameter being maintained at 15 mm at an inflation pressure of 8 atm. When the stricture site was still too narrow to allow colonoscope passage, balloon dilation was repeated until this was achieved (Fig. 2). After the procedure, patients routinely had plain abdominal X-ray examinations to rule out perforation and resumed their regular diet when no complications were observed. Those with a diverting stoma had routine follow-up colonoscopy after 2 weeks for outcome evaluation. Patients without a diverting stoma had clinical symptom evaluation to determine whether the procedure was successful.

## Results

There were 40 patients, (22 males and 18 females, mean age 64.6 ± 10.7 years range 33–84 years,). The median follow-up period was 56 months [interquartile range (IQR) 22.5–99 months]. The median time at which symptoms occurred after previous surgery was 7 months (IQR 5–11 months). The index operation was colorectal resection for malignant disease in 37/40 patients, the remaining 3 patients had been operated on for diverticulitis (*n* = 1), Crohn's disease (*n* = 1), and ulcerative colitis (*n* = 1). A total of 27 patients, (24 with malignant neoplasms and 3 with benign disease), had a diverting stoma requiring postoperative colonoscopy to identify and define the anastomotic stricture. Among those with malignant neoplasms, 13 (35%) received preoperative (*n* = 8) and postoperative (*n* = 5) radiation therapy. The mean stricture level was 7.1 ± 4.4 cm AAV. Ten patients had strictures 8 cm AAV, while the remaining 30 patients had strictures within 8 cm AA. All had failed initial finger dilation. Stapled anastomosis was the most frequently utilized initial anastomosis method (82.5%; *n* = 33).Only 7 patients (17.5%) received a handsewn anastomosis. Initial anastomotic leakage was observed in 4 patients (10%). The basic demographic characteristics of the patients are shown in Table 1.

**Table 1:** Demographics and perioperative data

Total patients	*N* = 40
Sex (M:F)	22:18
Age (years) mean, SD	64.6 ± 10.7
Primary disease	Cancer: 37 Benign: 3
Anastomosis level cm AAV	7.1 ± 4.4 ≥ 8 (*n* = 10) < 8 cm (*n* = 30)
Type of anastomosis (Stapled:HS)	33:7
Diverting ostomy (yes:no)	27:13
Radiotherapy (yes:no)	13:27 PreoP: postoP: 8:5
Anastomotic leak (yes:no)	4:36

*HS *hand-sewn, *AAV *above the anal verge

The initial success rate of balloon dilation was 100%. The results are provided in Table 2. The median operative time was 45 min (IQR 25–80 min). Only 1 procedure-related complication was observed (i.e., colon perforation). The perforation, which was caused by initially inserting the balloon catheter too far without withdrawing the guide wire, was approximately 10–20 cm proximal to the stricture lesion. This complication was identified during routine follow-up abdominal X-ray after the procedure. The patient received conservative treatment, including empiric antibiotics and cessation of enteric intake. Given that the patient already had a diverting colostomy, no intra-abdominal abscess developed. This patient underwent another session of balloon dilation combined with colostomy reversal 1 month after the first episode. A total of 7 (17.5%) recurrences were observed with a median time to recurrence of 3 months (IQR 2–8 months). Among those with a recurrence, 5 had another attempt at endoscopic balloon dilation and 2 had a total of 3 sessions. All 40 patients were symptom-free, had no imaging findings suggesting restenosis during the follow-up period, and did not need more aggressive management, such as redo anastomosis.

**Table 2:** Results

Total patients	*N* = 40
Primary success, *n* (%)	40 (100)
Time symptoms occurred, months median, (IQR)	7 (5–11)
Operation time, minutes, median (IQR)	45 (25–80) min
Complications	1 (2.5%)
Length of stay^a^, median(IQR)	1 (1–1) day
Recurrence	7 (17.5%)
Time to recurrence, months, median (IQR)	3 (2–8) m
Management of recurrence	5 patients had re-dilatation 2 patients had 3 sessions

*IQR* intraquartile range, Q1–Q3

^a^The length of stay was defined as hospital stay after operation. Combined surgery cases were excluded

We examined possible factors influencing recurrence, including age, sex, anastomosis type, anastomosis level, radiation therapy, diverting ostomy, and previous anastomotic leakage. None of the aforementioned factors were significantly associated with recurrence on Fisher's exact test (Table 3).

**Table 3:** Perioperative risk factors

Factor	Symptom free	Recurrence	*p* value
Age, years			
≤ 70	25	4	0.3694
> 70	8	3	
Sex			
Male	16	6	0.1048
Female	17	1	
Type of anastomosis			
Stapled	29	4	0.0877
HS	4	3	
Anastomosis level (cm, AAV)			
≥ 8	9	1	0.656
< 8	24	6	
Radiation therapy			
Yes	9	4	0.1866
No	24	3	
Ostomy			
Yes	22	5	1.0000
No	11	2	
Anastomotic leakage			
Yes	3	1	0.5522
No	30	6	

*HS *hand-sewn, *AAV *above the anal verge

In this study, 27 of the 40 patients had diverting stoma at initial operation. All our patients had colonoscopy before stoma reversal. There were 3 patients who had no stenosis at preoperative colonoscopy. These 3 patients developed stenosis at the initial anastomotic site not the stoma reversal site, after stoma reversal. After balloon dilation, 2 developed recurrence and  had a second dilation. A total of 22 patients had stoma reversal after the dilation procedure. Only 1 of these 22 patients developed recurrence 6 months after stoma reversal surgery, and he was treated with re-intervention with balloon dilation and has remained symptom-free until the time of writing (10 months). The median interval between the first balloon dilation procedure and stoma reversal surgery was 18.5 days (IQR 5–73 days). Ultimately, 2 patients did not undergo stoma reversal. Given that both patients received systemic chemotherapy for colorectal cancer metastasis and had a poor response, they continued chemotherapy and did not undergo stoma reversal surgery.

Combined surgery, (balloon dilatation and an additional operation done at the same time) was performed in 6 patients: 5 patients had balloon dilatation for stenosis and stoma reversal at the same time; the other one had balloon dilatation for stenosis and abdominal wall incisional hernia repair. No associated complications were noted. Median hospital stay after the procedure was 1 day (range 1–7 days; IQR 1–1 days). Excluding those who underwent combined surgery, the mean hospital stay after the procedure was 1.4 ± 1.2 days.

## Discussion

Studies haves shown that 3–30% of patients develop benign anastomotic strictures following colorectal surgery [2, 3]. Our preliminary study found an incidence rate of 3.14% [12], which was as low as previous studies. Anastomotic strictures have previously been observed at a median of 5–12 months following colorectal surgery [3, 13]. In the present study, anastomotic strictures occurred at a median of 7 months from the index surgery. However, anastomotic strictures in 24 patients who had a diverting stoma had been identified during scheduled follow-up colonoscopy. Therefore, occurrence times may have been overestimated given that such patients exhibited no symptoms. After excluding those with a diverting stoma, the median occurrence time decreased to 6.5 months (IQR 4–16 months) after the previous index surgery. The time at which symptom occurred in the current series was similar to that presented in previous studies. Accordingly, anastomotic stricture should be considered among patients who begin to complain of stool passage difficulty half a year after the index surgery.

Several techniques have been used to manage colorectal anastomotic strictures, such as surgical re-anastomosis, stents, cutter stapler devices, local steroid injections, dilation combined with electrocautery or laser photoablation, manual or instrumental balloon dilation under through-the-scope or fluoroscopic guidance [2, 13–15]. Previous studies have demonstrated that balloon dilation was a safe and effective procedure, with short-term recurrence rates ranging from 6–20% [16, 17]. The largest series, which followed 76 patients for a median of 17 years, revealed a long-term success rate of 75% [3]. The present study had observed similar good outcomes, with a 100% primary success rate and a 17.5% recurrence rate. Most recurrences observed herein occurred during first 3 months. Only one patient developed a recurrence after 14 months, while another developed a recurrence after 8 months. All recurrences were treated with repeat balloon dilation and remained symptom-free for a median follow-up duration of 5 years (IQR 2–7.5 years). Our experience suggests that using multiple diameter balloon could promote better primary success given that it gradually dilates the stricture site with steady pressure. We had dilated the stricture site using different balloon diameters at steady pressure to maintain the width of the lumen. Moreover, stricture recurrences can be managed using repeat dilation with good responses. The current series revealed that the procedure was considerably safe. One complication related to guided wire injury had occurred, which had been treated conservatively. Despite the safety of the procedure, abdominal X-ray is recommended after the procedure. Accordingly, perforations suspected from imaging findings should necessitate early treatment. Bleeding can be another common complication following balloon dilation [9, 18], although no such complication had been observed herein. Given that through-the-scope balloons for dilation, we were able to directly assess the dilated lesion through the scope. As such, oozing lesions could be compressed with the inflated balloon when observed and the balloon and colonoscope can be withdrawn after the oozing stops. That may explain why none of our patients experienced bleeding despite it being the second most common complication.

Given that stool passage may promote daily dilation following balloon dilation, stoma reversal was attempted as soon as possible among those with a diverting stoma, with the median interval between last balloon dilation procedure and stoma reversal surgery being 16 days (IQR 2–27 days). The presence of stricture lesions was assessed prior to stoma reversal. Accordingly, repeat balloon dilation instead of stoma reversal surgery was performed when stricture recurrence was observed. Most patients underwent stoma reversal surgery within 1 month. As our experience increased, we were able to simultaneously perform stoma reversal surgery sand balloon dilation in five cases without any complications. After the surgery, stool passage may promote daily dilation of the stricture, thereby preserving its patency. Only one case developed restenosis after balloon dilation and stoma reversal surgery. The aforementioned case exhibited symptoms 6 months after stoma reversal surgery, which were confirmed through colonoscopy. The patient subsequently underwent re-dilation and remained symptom free until present (10 months after the last dilation).

One study had shown that handsewn anastomosis is a risk factor for stricture recurrence [3]. Similarly, the present series found that handsewn anastomosis tended to promote higher recurrence rates compared to other techniques, albeit not significantly. Although Biraima et al. suggested that this finding may perhaps be related to low-lying anastomosis [3], the present study found no correlation between anastomotic level and stricture recurrence. No significant association was observed between other factors, such as previous anastomotic leakage or radiation therapy, which may worsen local fibrosis, and recurrence. Nonetheless, more sessions of balloon dilation may be needed to address problems related to handsewn anastomotic strictures.

Some limitations of the current study are worth noting. First, this was a retrospective study including a small number of patients. Second, endoscopic multidiameter balloon dilation was only applied to those with benign strictures, excluding those with suspicious lesions. Therefore, this procedure may not be suitable for complex cases.

## Conclusions

Endoscopic multidiameter balloon dilation is a safe and effective method for benign colorectal anastomosis strictures. Moreover, primary success rates were high, while the procedure can be easily repeated for recurrent cases. However, endoscopic multidiameter balloon dilation had higher failure rates for handsewn anastomosis strictures, which may require several sessions of dilation. Furthermore, our results suggested that simultaneous surgery is feasible and safe for stoma reversal.
